# A comparative study of salivary biomarkers for periodontal disease among smokers and non-smokers

**DOI:** 10.6026/973206300222828

**Published:** 2026-05-31

**Authors:** Anusha Pulluri, Saloni Tomar, Nabiha Naureen Syeda, Florence K Priya, Vaz Rachel Reemal, Naseemoon Shaik

**Affiliations:** 1 Department of Dentistry, Sri Sai College of Dental Surgery, Moinabad, Telangana, India; 2 Department of Dentistry, Kennedy Dental Center, Union City, NJ 07087, USA; 3 Department of Dentistry, Sri Balaji Dental College, Moinabad, Hyderabad, Telangana, India; 4 Department of Public Health Dentistry, Neuro and Dental Care, Secunderabad, Telangana, India; 5 Department of Dentistry, KVG Dental College Hospital, Sullia, Karnataka, India; 6 Department of Pedodontics and Preventive Dentistry, MNR Dental College and Hospital, Sangareddy, Telangana, India

**Keywords:** Salivary biomarkers, periodontal disease, smoking, oxidative stress, inflammatory cytokines

## Abstract

There is a need to understand how smoking alters salivary biomarkers and periodontal disease severity. Therefore, it is of interest to evaluate salivary biomarkers in smokers and non-smokers with chronic periodontitis. Hence, a cross-sectional design included 100 subjects, with clinical parameters and salivary biomarkers analyzed using ELISA and SPSS. Smokers showed significantly higher periodontal parameters and elevated inflammatory and oxidative stress markers, with reduced antioxidant levels. Thus, we show salivary biomarkers as reliable non-invasive indicators for monitoring periodontal disease, especially in smokers. Salivary biomarkers can serve as effective, non-invasive tools for diagnosis and disease monitoring, particularly in smokers.

## Background:

Periodontal disease is a chronic inflammatory condition affecting the supporting structures of the teeth, including the gingiva, periodontal ligament, cementum and alveolar bone [1]. It is one of the most prevalent oral diseases world wide and represents a major cause of tooth loss in adults. The pathogenesis of periodontal disease is multifactorial, involving a complex interplay between microbial biofilms and the host immune-inflammatory response. While dental plaque serves as the primary etiological factor, the severity and progression of the disease are significantly influenced by various local and systemic risk factors, among which tobacco smoking is considered one of the most critical [2]. Smoking has long been recognized as a major environmental risk factor that adversely affects periodontal health. Numerous epidemiological and clinical studies have demonstrated a higher prevalence, severity and extent of periodontal destruction among smokers compared to non-smokers [3]. Smokers exhibit deeper periodontal pockets, greater clinical attachment loss and increased alveolar bone resorption. Additionally, smoking impairs healing following periodontal therapy and contributes to a poorer prognosis. The deleterious effects of smoking are attributed to its impact on vascularity, immune response and cellular functions, leading to altered host-microbial interactions [4]. The biological mechanisms underlying smoking-induced periodontal damage are complex. Tobacco smoke contains a multitude of harmful chemicals, including nicotine, carbon monoxide and reactive oxygen species, which contribute to oxidative stress and tissue injury. Smoking compromises neutrophil function alters cytokine production and reduces antibody responses, thereby impairing the host's ability to combat periodontal pathogens effectively. Furthermore, it leads to vasoconstriction and reduced blood flow to the gingival tissues, masking clinical signs of inflammation such as bleeding on probing, which can delay diagnosis and treatment [5].

In recent years, there has been growing interest in the use of salivary biomarkers as diagnostic and prognostic tools in periodontal disease. Saliva is an easily accessible, non-invasive biological fluid that contains a wide array of constituents, including enzymes, cytokines, immunoglobulin and oxidative stress markers. These components reflect both local and systemic physiological and pathological conditions, making saliva a valuable medium for disease detection and monitoring [6]. Unlike traditional clinical parameters, which primarily assess past disease activity, salivary biomarkers offer the potential for real-time evaluation of ongoing disease processes. Several salivary biomarkers have been studied in relation to periodontal disease. Pro-inflammatory cytokines such as interleukin-1 beta (IL-1β) , tumor necrosis factor-alpha (TNF-α) and interleukin-6 (IL-6) are known to play pivotal roles in the inflammatory cascade and tissue destruction associated with periodontitis [7]. Enzymes such as matrix metalloproteinases (MMPs), particularly MMP-8 and MMP-9, are involved in the degradation of extracellular matrix components and are considered key indicators of periodontal tissue breakdown. Additionally, markers of oxidative stress, such as malondialdehyde (MDA) and antioxidant enzymes like superoxide dismutase (SOD) have been investigated to understand the balance between oxidative damage and defense mechanisms in periodontal disease. The influence of smoking on salivary biomarkers is of particular interest, as tobacco use may alter the expression and levels of these biomarkers, thereby affecting disease progression and diagnostic interpretation [8]. Studies have suggested that smokers exhibit elevated levels of pro-inflammatory cytokines and oxidative stress markers, along with reduced antioxidant capacity, compared to non-smokers. However, the exact patterns and clinical significance of these alterations remain inconsistent across studies. This variability highlights the need for further research to establish reliable biomarker profiles that can differentiate between smokers and non-smokers with periodontal disease. Comparative evaluation of salivary biomarkers in smokers and non-smokers with periodontal disease can provide valuable insights into the pathophysiological differences influenced by tobacco exposure. It can also aid in identifying specific biomarkers that are more sensitive or specific in detecting disease activity in these populations. Such information is crucial for developing personalized diagnostic approaches and targeted therapeutic strategies. Moreover, understanding these differences may contribute to improved risk assessment, early detection and better management of periodontal disease in smokers [9]. Despite advancements in periodontal diagnostics, there remains a need for non-invasive, cost-effective and reliable methods to assess disease activity and progression. Salivary diagnostics holds promise in fulfilling this need, particularly in large-scale screenings and routine clinical practice [10]. Therefore, it is of interest to determine the variations in salivary biomarker levels between smokers and non-smokers with periodontal disease and to assess their potential role in disease diagnosis, progression and management.

## Methodology:

## Study design and population:

This study was designed as a cross-sectional comparative analytical study to evaluate salivary biomarkers in smokers and non-smokers with periodontal disease. A total of 100 subjects were recruited from patients reporting to the Department of Periodontology. The study population was divided into two groups: Group I (smokers with periodontal disease, n = 50) and Group II (non-smokers with periodontal disease, n = 50).

## Inclusion criteria:

Participants aged between 25-55 years diagnosed with chronic periodontitis were included in the study. Diagnosis was based on clinical parameters such as probing pocket depth (PPD) ≥4 mm and clinical attachment loss (CAL) ≥3 mm in at least two non-adjacent teeth. Subjects in the smoker group had a history of smoking at least 10 cigarettes per day for a minimum of 2 years. Non-smokers were individuals who had never smoked.

## Exclusion criteria:

Subjects with systemic diseases such as diabetes mellitus, cardiovascular disorders, or immunocompromised conditions were excluded. Individuals who had received periodontal therapy or antibiotics in the past 6 months, pregnant or lactating women, alcohol users and patients using smokeless tobacco were also excluded to avoid confounding factors.

## Ethical considerations:

The study protocol was reviewed and approved by the Institutional Ethical Committee. Written informed consent was obtained from all participants prior to inclusion in the study and the study was conducted in accordance with ethical standards.

## Clinical periodontal examination:

All subjects underwent a comprehensive periodontal examination performed by a single calibrated examiner to eliminate inter-examiner variability. Clinical parameters recorded included Plaque Index (PI), Gingival Index (GI), Probing Pocket Depth (PPD) and Clinical Attachment Level (CAL). Measurements were taken at six sites per tooth using a standardized periodontal probe.

## Saliva collection:

Unstimulated whole saliva samples were collected from all participants between 9:00 AM and 11:00 AM to minimize diurnal variations. Subjects were instructed to refrain from eating, drinking, or oral hygiene procedures for at least 1 hour prior to sample collection. Approximately 5 mL of saliva was collected by passive drooling into sterile containers. The samples were immediately stored at-20°C until biochemical analysis.

## Biochemical analysis of salivary biomarkers:

Salivary samples were centrifuged at 3000 rpm for 10 minutes to remove debris. The supernatant was used for the estimation of biomarkers. The levels of pro-inflammatory cytokines such as Interleukin-1β (IL-1β), Tumor Necrosis Factor-α (TNF-α)and Interleukin-6 (IL-6) were measured using enzyme-linked immunosorbent assay (ELISA). Matrix metalloproteinase-8 (MMP-8) levels were also assessed as a marker of tissue destruction. Oxidative stress was evaluated by estimating malondialdehyde (MDA) levels, while antioxidant status was determined by measuring superoxide dismutase (SOD) activity.

## Statistical analysis:

The collected data were entered into Microsoft Excel and analyzed using Statistical Package for Social Sciences (SPSS) version 25.0. Descriptive statistics were expressed as mean and standard deviation. Intergroup comparisons between smokers and non-smokers were performed using independent t-test for normally distributed data and Mann-Whitney U test for non-parametric data. Correlation between clinical parameters and salivary biomarkers was assessed using Pearson's correlation coefficient. A p-value of <0.05 was considered statistically significant.

## Sample size justification:

A total sample size of 100 subjects (50 per group) was considered adequate to achieve sufficient statistical power based on previous similar studies evaluating salivary biomarkers in periodontal disease, allowing for reliable comparison between smokers and non-smokers.

## Results:

A total of 100 subjects were included in the study, comprising 50 smokers and 50 non-smokers with periodontal disease. Gender distribution was comparable between the groups. Clinical periodontal parameters demonstrated significantly greater disease severity in smokers compared to non-smokers. The mean Plaque Index (PI), Gingival Index (GI), Probing Pocket Depth (PPD) and Clinical Attachment Level (CAL) were higher in smokers, indicating more advanced periodontal destruction (Table 1). Salivary biomarker analysis revealed significantly elevated levels of pro-inflammatory cytokines (IL-1β, TNF-α, IL-6) in smokers compared to non-smokers (Table 2). This suggests an enhanced inflammatory response in smokers with periodontal disease. Matrix metalloproteinase-8 (MMP-8), a marker of periodontal tissue breakdown, was significantly higher in smokers, indicating increased collagen degradation and disease activity (Table 3). Oxidative stress markers showed that malondialdehyde (MDA) levels were significantly higher in smokers, whereas antioxidant enzyme superoxide dismutase (SOD) levels were significantly lower compared to non-smokers (Table 4). This indicates increased oxidative stress and compromised antioxidant defense in smokers. Correlation analysis demonstrated a strong positive correlation between salivary biomarkers and clinical periodontal parameters in smokers. IL-1β, TNF-α and MMP-8 showed significant positive correlation with PPD and CAL, while SOD showed a negative correlation (Table 5). Overall, the results indicate that smokers with periodontal disease exhibit significantly worse clinical parameters, elevated inflammatory and oxidative stress markers and reduced a ntioxidant levels compared to non-smokers, highlighting the detrimental impact of smoking on periodontal health. Independent t-test revealed statistically significant differences between smokers and non-smokers for all clinical parameters (PI, GI and PPD, CAL) with p < 0.01. Salivary biomarkers including IL-1β, TNF-α, IL-6, MMP-8 and MDA were significantly elevated in smokers (p < 0.001), whereas SOD levels were significantly reduced (p < 0.001). Pearson correlation analysis showed strong positive correlations between inflammatory biomarkers and periodontal destruction parameters (PPD and CAL), particularly in smokers. Regression analysis indicated that MMP-8 and IL-1β were the strongest predictors of periodontal disease severity (β = 0.48 and β = 0.42 respectively, p < 0.001).

## Discussion:

The present study evaluated and compared salivary biomarkers in smokers and non-smokers with periodontal disease, revealing significantly higher levels of pro-inflammatory cytokines (IL-1β, TNF-α, IL-6), matrix metalloproteinase-8 (MMP-8) and oxidative stress marker (MDA) in smokers, along with reduced antioxidant levels (SOD). These findings indicate that smoking exacerbates periodontal inflammation and tissue destruction through modulation of host immune response and oxidative stress pathways. The elevated levels of IL-1β and TNF-α observed in smokers in the present study are consistent with the findings of Babayigit *et al.* (2026) [11] who reported significantly increased salivary IL-1β and TNF-α levels in periodontitis patients and highlighted the influence of smoking on biomarker expression. Their study also demonstrated a strong association between TNF-α levels and clinical periodontal parameters, which aligns with our findings showing positive correlations with PPD and CAL. Similarly, the findings of the present study are in agreement with Singh *et al.* (2014) [12] who reported elevated salivary TNF-α level in smokers with chronic periodontitis. Their study emphasized the role of TNF-α in mediating periodontal inflammation and tissue destruction. Our results further strengthen this evidence by demonstrating statistically significant differences between smokers and non-smokers across multiple inflammatory biomarkers. The increase in IL-1β levels observed in this study is also supported by Sánchez *et al.* (2013) [13] who demonstrated that salivary IL-1β levels increase proportionally with the severity of periodontal disease and can serve as a sensitive diagnostic marker. This is consistent with our findings, where higher IL-1β levels were associated with greater clinical attachment loss and probing depth in smokers. Further support is provided by Gursoy *et al.* (2009) [14] who identified IL-1β as a key biomarker reflecting both microbial burden and host inflammatory response in periodontitis. Our study expands on these findings by showing that smoking significantly amplifies IL-1β expression, suggesting a compounded effect of tobacco exposure on periodontal inflammation. In addition, the role of cytokines as reliable biomarkers is well supported by Gomes *et al.* (2016) [15] & Dhulipalla *et al.* (2025) [16] who concluded that IL-1β and TNF-α levels are significantly associated with periodontal disease severity and decrease following periodontal therapy. This aligns with the present study, where elevated cytokine levels were directly correlated with worsening clinical periodontal parameters. Apart from inflammatory biomarkers, the present study also demonstrated significantly higher oxidative stress levels (MDA) and reduced antioxidant capacity (SOD) in smokers. These findings indicate that smoking induces an imbalance between oxidants and antioxidants, leading to enhanced tissue destruction. Additionally, increased MMP-8 levels in smokers further confirm accelerated collagen degradation and periodontal breakdown, supporting the concept that smoking enhances proteolytic activity within periodontal tissues. Overall, the findings of the present study are in strong agreement with previous literature, confirming that salivary biomarkers such as IL-1β and TNF-α are elevated in periodontal disease and are further influenced by smoking. However, our study provides a more comprehensive evaluation by incorporating oxidative stress and tissue destruction markers alongside inflammatory cytokines, thereby offering a broader understanding of disease mechanisms. Variations between studies may be attributed to differences in study design, sample size, population characteristics and biomarker analysis techniques. Nevertheless, the consistent trend across the selected studies supports the validity of salivary biomarkers as reliable diagnostic tools and underscores the detrimental impact of smoking on periodontal health. Thus, the present study not only corroborates existing evidence but also emphasizes the importance of salivary biomarker assessment in improving early diagnosis, disease monitoring and personalized management of periodontal disease, particularly in smokers.

## Conclusion:

Smoking significantly exacerbates periodontal disease by increasing inflammatory and oxidative stress biomarkers. Smokers showed higher levels of IL-1β, TNF-α, MMP-8 and MDA, along with reduced antioxidant capacity (SOD). These alterations were strongly associated with increased periodontal destruction and disease severity. Salivary biomarkers proved to be reliable, non-invasive indicators for assessing periodontal status. Thus, salivary biomarker analysis can aid in early diagnosis and personalized management of periodontal disease, especially in smokers.

## Figures and Tables

**Table 1:** Comparison of clinical periodontal parameters between smokers and non-smokers

**Parameter**	**Smokers (Mean ± SD)**	**Non-Smokers (Mean ± SD)**	**p-value**
Plaque Index (PI)	2.31 ± 0.42	1.98 ± 0.36	0.001*
Gingival Index (GI)	2.12 ± 0.39	1.85 ± 0.33	0.002*
Probing Pocket Depth (PPD) (mm)	5.42 ± 0.78	4.63 ± 0.65	<0.001*
Clinical Attachment Level (CAL) (mm)	5.96 ± 0.84	5.02 ± 0.71	<0.001*
(*Statistically significant)

**Table 2:** Salivary Pro-inflammatory cytokines comparison of smokers and non-smokers

**Biomarker**	**Smokers (Mean ± SD)**	**Non-Smokers (Mean ± SD)**	**p-value**
IL-1β (pg/mL)	145.6 ± 22.4	118.3 ± 19.7	<0.001*
TNF-α (pg/mL)	98.7 ± 15.2	79.5 ± 13.6	<0.001*
IL-6 (pg/mL)	72.4 ± 11.8	60.2 ± 10.5	0.001*
(*Statistically significant)

**Table 3:** Comparison of MMP-8 Levels

**Biomarker**	**Smokers (Mean ± SD)**	**Non-Smokers (Mean ± SD)**	**p-value**
MMP-8 (ng/mL)	312.5 ± 45.6	268.4 ± 39.2	<0.001*
(*Statistically significant)

**Table 4:** Comparison of oxidative stress and antioxidant markers

**Biomarker**	**Smokers (Mean ± SD)**	**Non-Smokers (Mean ± SD)**	**p-value**
MDA (nmol/mL)	5.82 ± 0.94	4.36 ± 0.81	<0.001*
SOD (U/mL)	1.92 ± 0.34	2.48 ± 0.41	<0.001*
(*Statistically significant)

**Table 5:** Correlation between salivary biomarkers and clinical parameters (Smokers group)

**Biomarker**	**PPD (r-value)**	**CAL (r-value)**	**p-value**
IL-1β	0.62	0.58	<0.001*
TNF-α	0.55	0.52	0.002*
MMP-8	0.67	0.63	<0.001*
(*Statistically significant)
